# Echinacoside reverses myocardial remodeling and improves heart function via regulating SIRT1/FOXO3a/MnSOD axis in HF rats induced by isoproterenol

**DOI:** 10.1111/jcmm.15904

**Published:** 2020-12-13

**Authors:** Yajuan Ni, Jie Deng, Xin Liu, Qing Li, Juanli Zhang, Hongyuan Bai, Jingwen Zhang

**Affiliations:** ^1^ Department of Cardiology The Second Affiliated Hospital of Xi’an Jiaotong University Xi'an shaanxi China; ^2^ Department of Cardiology, NHC Key Laboratory on Assisted Circulation of the First Affiliated Hospital Sun Yat‐sen University Guangzhou Guangdong China

**Keywords:** echinacoside, heart failure, mitochondrial oxidative stress, myocardial remodelling

## Abstract

Myocardial remodelling is important pathological basis of HF, mitochondrial oxidative stress is a promoter to myocardial hypertrophy, fibrosis and apoptosis. ECH is the major active component of a traditional Chinese medicine Cistanches Herba, plenty of studies indicate it possesses a strong antioxidant capacity in nerve cells and tumour, it inhibits mitochondrial oxidative stress, protects mitochondrial function, but the specific mechanism is unclear. SIRT1/FOXO3a/MnSOD is an important antioxidant axis, study finds that ECH binds covalently to SIRT1 as a ligand and up‐regulates the expression of SIRT1 in brain cells. We hypothesizes that ECH may reverse myocardial remodelling and improve heart function of HF via regulating SIRT1/FOXO3a/MnSOD signalling axis and inhibit mitochondrial oxidative stress in cardiomyocytes. Here, we firstly induce cellular model of oxidative stress by ISO with AC‐16 cells and pre‐treat with ECH, the level of mitochondrial ROS, mtDNA oxidative injury, MMP, carbonylated protein, lipid peroxidation, intracellular ROS and apoptosis are detected, confirm the effect of ECH in mitochondrial oxidative stress and function in vitro. Then, we establish a HF rat model induced by ISO and pre‐treat with ECH. Indexes of heart function, myocardial remodelling, mitochondrial oxidative stress and function, expression of SIRT1/FOXO3a/MnSOD signalling axis are measured, the data indicate that ECH improves heart function, inhibits myocardial hypertrophy, fibrosis and apoptosis, increases the expression of SIRT1/FOXO3a/MnSOD signalling axis, reduces the mitochondrial oxidative damages, protects mitochondrial function. We conclude that ECH reverses myocardial remodelling and improves cardiac function via up‐regulating SIRT1/FOXO3a/MnSOD axis and inhibiting mitochondrial oxidative stress in HF rats.

## 1 INTRODUCTION

Heart failure (HF) is the ultimate outcome of most cardiovascular diseases with high prevalence and poor prognosis, and with the aggravating trend of an ageing population, the hospitalization and mortality due to chronic heart failure (CHF) increased sharply all around the world.^1^ During the past 20 years, drug treatments for HF have made great progress, but the effect is very limited, so new drugs are urgently needed. Myocardial remodelling is important pathological basis of HF and is characterized by myocardial hypertrophy, apoptosis, interstitial fibrosis, the myocardial cells are disorganized, and lead to reduction of ejection fraction (LVEF), increase of left ventricular end‐diastolic and systolic dimensions (LVIDd and LVIDs), and decrease of left ventricular fractional shortening (LVFS).^2^ But the mechanisms underlying were still not fully understood. In recent years, numerous studies have confirmed that oxidative stress is an important promoter of myocardial remodelling.^3^, ^4^, ^5^, ^6^, ^7^

Oxidative stress is a condition in which reactive oxygen species (ROS) or free radicals are generated excessively and lead to oxidative damage of cell components, this in turn causes cell dysfunction.^8^ Mitochondria are the main source of intracellular ROS, as a result of leakage from the respiratory electron transport chain, and mitochondria are the most sensitive position about the ROS' effects.^9^ Studies in HF identified increased ROS generation from complex I of the respiratory chain.^8^ Increased ROS production attacks mitochondrial component firstly and results in excess oxidative stress, manifests as carbonylation of proteins, lipid peroxidation, and DNA damage,^10^ and dysfunction of mitochondria, which consequently lead to excessive formation of ROS,^8^ forms a vicious circle. The sustained oxidative stress is demonstrated can cause myocardial hypertrophy and myocardial fibrosis,^11^, ^12^, ^13^ and cause apoptosis of myocardial cells through activation of mitochondria‐dependent apoptosis as a result of carbonylation of mitochondrial membrane proteins and loss of mitochondrial membrane potential.^14^ So, it is considered that inhibition of mitochondrial oxidative stress may be a new strategy to prevent myocardial remodelling and even HF.^15^, ^16^, ^17^

Echinacoside (ECH), a natural phenylethanoid glycoside, is the major active component of Cistanches Herba, which is a traditional Chinese medicine. In recent years, ECH has been extensively studied in nervous system and tumour, and it has been reported to possess a variety of pharmacological effects, such as antioxidant, anti‐inflammatory, anti‐apoptosis and anti‐tumour properties.^18^, ^19^ In nerve cells, ECH significantly enhances antioxidant capacity, reduces ROS production, improves mitochondrial membrane potential (MMP) and increases cell viability,^20^ exhibits protective effects on mitochondrial function and inhibits mitochondrial oxidative stress, it protects against oxidative injury and improves memory, eventually prevents neurodegeneration.^21^, ^22^ ECH also increases the activity of SOD and play a protective role in brain tissue and retina tissue,^19^, ^23^ but the specific mechanism for its antioxidant effect is unclear.

SIRT1 is closely related to oxidative stress, and SIRT1/FOXO3a/MnSOD signalling axis is the most important antioxidant axis and responsible for suppression of mitochondrial oxidative stress, MnSOD distributes widely in mitochondria and primarily responsible for clearing of mitochondrial ROS, thus inhibiting oxidative stress, compounds which activate this antioxidant axis can inhibit oxidative stress.^24^, ^25^ Recent studies have shown that ECH exerts it pharmacological effects via regulating multiple signalling pathways, such as p‐AKT, mTOR/STAT3, TGF‐beta 1/Smads and SIRT1 signalling pathway.^26^, ^27^, ^28^, ^29^ And a new study finds that ECH binds covalently to SIRT1 as a ligand and up‐regulates the expression of SIRT1 in brain cells.^26^ It suggests that ECH may inhibit mitochondrial oxidative stress in cardiomyocytes and reverse myocardial remodelling. However, researches on the effects of ECH have focused on the nervous system or tumour, the effects and mechanisms of ECH on mitochondrial oxidative stress in cardiomyocytes and myocardial remodelling remain unknown. Here, we firstly induce cellular model of oxidative stress by ISO and confirm the effect of ECH in mitochondrial oxidative stress and function in vitro. Then, we establish a HF rat model induced by ISO and demonstrate that ECH reverses myocardial remodelling and improves cardiac function via up‐regulating SIRT1/FOXO3a/MnSOD signalling axis and inhibiting mitochondrial oxidative stress in HF rats. The results are intending to provide experimental evidence for the development of new drugs to prevent heart failure.

## 2 MATERIALS AND METHODS

### 2.1 Cell culture and treatment

AC16 cells are purchased from BeNa Culture Collection. The cells are maintained in high glucose Dulbecco's modified Eagle's medium (DMEM; Hyclone, GE healthcare, USA) supplement with 10% foetal bovine serum are (10%FBS; Gibco) in T‐75 flasks (Corning Glassworks, Corning, NY, USA). Conditions are maintained at 37℃ in a humidified atmosphere containing 5% CO_2_. Once the cells reach 70%‐80% confluence, ISO (Sigma, USA) group cells are incubated with medium containing 10uM ISO (Sigma, USA) for 24h, ECH group cells are pre‐treated with 50uM ECH (MCE, USA) for 30min prior ISO exposure. Control cells (Ctrl) are treated nothing.

### 2.2 Detection of mitochondrial ROS level using luminol chemiluminescence

Mitochondria are isolated and purified of AC‐16 cells using Mitochondria Isolation Kit (Sigma, USA) according to the manufacturer's instructions. Protein concentrations are determined by BCA protein assay kit (Bio tech). Mitochondrial ROS level is detected by luminal (3‐aminophthalic hydrazide 5‐amino‐2,3‐dihvdro‐1,4‐phthalazinedione) using an Elisa kit (GENMED SCIENTIFICS INC,USA), it reacts with ROS and causes emission of photons, which is a band spectrum having a peak at about 460nm, and Relative Light Unit (RLU) is detected by chemiluminescence measuring instrument and converted into RLU/μg mitochondrial protein.

### 2.3 8‐OHdG is used to evaluate the oxidative damage of mitochondrial DNA

Mitochondrial DNA oxidative damage is evaluated by detecting the content of 8‐Hydroxy‐2‐deoxyguanosine (8‐OHdG). Purified mitochondria in cells are isolated using mitochondria isolation kit as above (Sigma). Protein concentrations are determined by BCA protein assay kit (Bio tech). Then, they are dissociated to determine the level of 8‐OHdG using an Elisa kit (GENMED SCIENTIFICS INC, USA) according to the manufacturer's instructions. The absorbance of the colour change is measured in spectrophotometer at the wavelength of 450 nm. The concentration of 8‐OHdG in the samples is then determined by comparing the OD of the samples to the standard curve, and converted into ng/ml.

### 2.4 Measurement of MMP by JC‐1

Mitochondria are isolated and purified of AC‐16 and protein concentration is determined as mentioned above. Then the mitochondria are resuspended at 1mg/ml concentration, the MMP is measured using JC‐1 assay kit according to the manufacturer's instructions. The fluorescence intensity is measured at excitation wavelength: 490nm, slit 5nm, emission wavelength: 590 nm, slit 5nm with a spectrophotometer instrument,^30^ the results are represent as FLU/μgP.

### 2.5 Detection of apoptotic cells by flow cytometry in AC‐16 Cells

Flow cytometry is used to assess cellular apoptosis. An Annexin V‐FITC/PI apoptosis detection kit (CWBIO, China) is used to detect apoptotic and necrotic cells according to the supplier's instructions. Early stage of apoptosis, late stage of apoptosis or necrosis, cellular debris and viable cells are identified as annexin V (+)/PI (−), annexin V (+)/PI (+), annexin V (−)/PI (+) and annexin V (−)/PI (−), respectively. Fluorescence intensities are analysed using a flow cytometer (BD FACSVerse™ Flow Cytometer, BD Biosciences, San Jose, CA, USA). Each experiment is repeated 3 times.

### 2.6 Assessed the intracellular ROS by flow cytometer

After treatment, cells are trypsinized, washed twice with PBS. The cells are then resuspended in PBS, stained with DCFH‐DA (Beyotime, China) at the final concentration of 1 μM and incubated for 1 h at 37℃ in an incubator with CO_2_, and then tested in a flow cytometer at 488 nm excitation. The fluorescence intensity represents the level of intracellular ROS.

### 2.7 Test carbonyl protein content in mitochondria of AC‐16 cells

After treatment, purified mitochondria are isolated and sonicated on ice and are treated with 1% streptomycin sulphate to precipitate mitochondrial nucleic acids, protein concentration are determined. The carbonyl protein content is tested using an Elisa kit (GENMED SCIENTIFICS INC, USA) based on derivatization of the carbonyl group with dinitrophenylhydrazine (DNPH), according to the instructions of the kit, OD is measured by a microplate reader and converted into nmol/mg protein by comparing to the standard curve.

### 2.8 Determination of lipid peroxidation level in mitochondria of AC‐16 cells

After treatment, cells are collected and lysed, mitochondria are isolated and purified and cracked by ultrasound, protein concentration is determined by BCA protein assay kit (Bio tech), lipid peroxidation is determined by the level of malondialdehyde (MDA) via the TBARS assay, using a kit (Nan jing jian cheng, China) and MDA level is measured following the manufacturer's protocol. OD is detected at 586 nm by a microplate reader and converted into μmol/g protein by comparing to the standard curve.

### 2.9 Animals and treatment

Young male Sprague‐Dawley rats weighing 120‐140g are acquired from Xi'an Jiao tong University Laboratorial Animal Center (Shaanxi, China). The investigation conforms to The Guide for the Care and Use of Laboratory Animals, published by the US National Institutes of Health (NIH publication no.85‐23, revised in 1996), and the experimental protocols were approved by the local authorities (Biomedical Ethics Committee of Medical Department of Xi 'an Jiao tong University). Rat model of HF is established as described previously by us.^31^ Briefly, Isoproterenol (10 mg/kg) is administered once daily by intraperitoneal injection for 2 weeks (defined as HF group), ECH is administered with 20 ug/g once daily by intraperitoneal injection at 30min before ISO is treated (defined as ECH group), and the administration lasts for 2 weeks. Control animals are administrated with 0.9% NaCl (defined as Ctrl group). Echocardiography measurements are performed to evaluate the heart function.

### 2.10 Echocardiographic measurements

Animals are anaesthetized by an intraperitoneal injection of 300 mg/kg of chloral hydrate. The SONOS‐2500 ultrasound system with an ultrasound transducer of 7.5 MHz (HP, USA) is used to make echocardiographic examinations. The left ventricular ejection fraction (LVEF), left ventricular fractional shortening (LVFS), left ventricular end‐diastolic dimensions (LVIDd), left ventricular end‐systolic dimensions (LVIDs), heart rate (HR), interventricular septal thickness in diastole (IVSTd), left ventricular posterior wall thickness in diastole (LVPWTd) are measured as described previously by us.^32^

### 2.11 Heart weight to body weight ratio (HW/BW) is measured

Rat is weighed before anaesthetization, fresh heart is isolated and wash out blood with 0.1mol/L PBS, cut away vessels and other redundant tissue and the heart is weighed, then HW/BW(mg/100g) is calculated.

### 2.12 Histological staining

The hearts of rats are dissected and fixed in 4% paraformaldehyde, embedded in paraffin and sectioned into 5 μm‐thick slices. Haematoxylin‐Eosin (HE) staining is used to observe the pathological changes and Masson's trichrome is used to evaluate collagen fibres of rats myocardium tissue. The cardiac collagen volume fraction (CVF) and cardiomyocytes cross‐sectional area is measured with Image‐Pro Plus 6.0 (Media Cybernetics, Bethesda, MD, USA).

### 2.13 TUNEL staining

Myocardial tissue from the left ventricle is collected and the TUNEL assay is carried out as described previously by us^32^

### 2.14 Transmission electron microscope

Fresh heart tissue is fixed in 2.5% glutaraldehyde at 4℃, tissue is then cutted into 1mm^3^ in volume and fixed again in 2.5% glutaraldehyde at 4℃ for 2h, then is washed with 0.1mol/L PBS, fixed in 1% osmic acid, dehydrate with acetone, embedded with epoxy resin, ultrathin slices which thin about 800nm are made, stained with uranyl acetate and lead citrate, and then are observed under a transmission electron microscope (H‐7650, Hitachi Limited, Japan). To quantify the protective effect of ECH in mitochondrial morphology, the mean mitochondrial circumference, mean mitochondrial cross‐sectional area, percentage of degeneration and percentage of vacuolization in mitochondria are analysed statistically with Image‐Pro Plus 6.0 (Media Cybernetics, USA).

### 2.15 Quantitative PCR

Total RNA is prepared from left ventricular tissue with Trizol reagent (Invitrogen, Carlsbad, USA). cDNA is synthesized using the TUREscript 1st Strand cDNA Synthesis Kit (Takara, Japan). Specific primer sequences of collagen I used for real‐time PCR is as follows: forward: 5′‐GTCGTATCCAGTGCGTGTC‐3′, revers: 5′‐GTGGAGTCGGCAATTGCA‐3′. GAPDH forward 5'‐GGC AAG GTC ATCCCA GAG CT‐3', reverse 5'‐CGC CTG CTT CAC CAC CTT CT‐3'.Real‐Time quantitative PCR (qPCR) is performed with SYBRPremix Ex Taq (Perfect Real Time) (Takara, Japan). The relative level of mRNA is calculated by normalizing to GAPDH, according to the 2^‐ΔΔCT^ method.

### 2.16 Western blot analysis of SIRT1, FOXO3a, MnSOD protein expression

Total protein of left ventricular tissue are extracted on ice using cold RIPA Buffer (BioRad, USA) with Protease Inhibitor Cocktail (Sigma‐Aldrich). The concentration is determined by BCA method. Procedures of Western blot are described previously.^33^ Briefly, protein sample is fractionated on 15% sodium dodecylsulphate‐polyacrylamide gels, transferred to PVDF membranes (BioRad, USA), blocked by 5% skim milk and incubated with MYH antibody from mouse (diluted 1:200, Santa Cruz, USA). Then, incubated with a horseradish peroxidase‐conjugated goat mouse antibody (diluted 1:2,000, Santa Cruz, USA). The relative level of protein is calculated by normalizing mean ray value to β‐actin. Protein bands are detected by a chemiluminescence system (ChemiDoc XRS, BioRad).

### 2.17 Measurement of mitochondrial oxidative stress in rat myocardium

Purified mitochondria of rat myocardium are isolated using Mitochondria Isolation Kit (Sigma, USA) according to the manufacturer's instructions, mitochondrial ROS, MMP, oxidative damage of mtDNA, carbonylated protein content and lipid peroxidation level in mitochondria are all detected using the same methods as cell experiments described above. Of note, ROS in myocardial tissue is detected with DCFH‐DA fluorescent probes, fluorescence intensity is detected with enzyme‐labelling measuring instrument, the results are represented as RLU/μg mitochondrial protein.

### 2.18 Statistical analysis

All data are presented as means ± SEM. One‐way ANOVA is used to compare differences among multiple groups, followed by Tukey post hoc test for significance. All statistics are determined using SPSS15.0 software (SPSS Inc, IL, USA). A probability value of *P* < .05 is considered significant.

## 3 RESULTS

### 3.1 50 μM ECH effectively inhibits mitochondrial oxidative damage and apoptosis in AC‐16 cells

**Figure 1:**
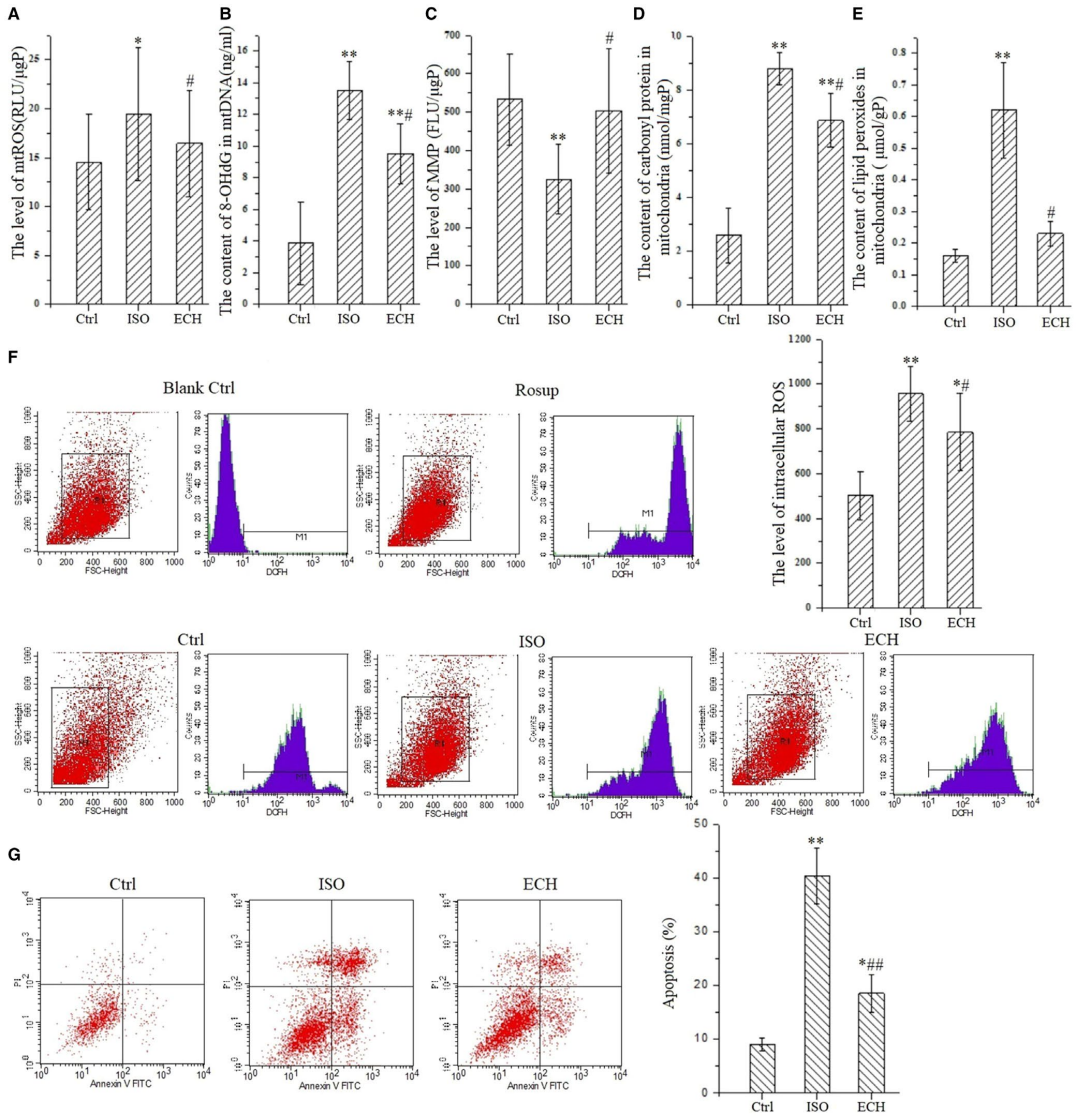
50μmol ECH effectively inhibits mitochondrial oxidative stress of AC‐16 cells induced by 10μmol/L ISO and protects mitochondrial function and reduces apoptosis. A, ECH reduces the release of mitochondrial ROS induced by ISO, y represents the relative light unit (RLU) per μg mitochondrial protein. B, ECH inhibits oxidative damage of mtDNA induced by ISO, y represents the level of 8‐OHdG. C, ECH protects MMP damaged by ISO, y represents the fluorescence intensity per μg mitochondrial protein. D, ECH decreases carbonyl protein content of mitochondria induced by ISO, y represents the level of carbonyl protein per mg mitochondrial protein. E, ECH decreases lipid peroxidation level of mitochondria induced by ISO, y represents the level of lipid peroxides MDA per g mitochondrial protein. F, Results of flow cytometry show that ECH reduces the accumulation of intracellular ROS in AC‐16 cells induced by ISO. G, Results of flow cytometry show that ECH intensively suppresses cell apoptosis. All *P < .05 versus Ctrl,**P < .01 versus Ctrl; *^#^P* < .05 versus ISO, *^##^P* < .01 versus ISO. Error bars represent SD

Untreated cells are set as Ctrl group. Firstly, as Figure 1A shows, ECH inhibits mitochondrial ROS induced by ISO. ISO treated cells shows a significant increase of mitochondrial ROS compared with Ctrl and this increase is significantly attenuated by pre‐treatment with 50 μM ECH. Secondly, ECH can inhibit oxidative damage of mtDNA induced by ISO. 8‐OHdG is believed to be one of the most abundant DNA lesions resulting from oxidative stress and is a biomarker of the oxidative DNA damage and repair. As Figure 1B shows, the content of 8‐OHdG in purified mitochondria from AC‐16 cells is detected, the results show that the level of 8‐OHdG in mitochondria from ISO treated cells dramatically increased, but the change was reversed by 50 μM ECH. Thirdly, ECH is effective to protect the MMP, which is impaired by ISO. The fluorescence intensity is significantly decreased in cells treated with ISO compared with Ctrl cells, whereas pre‐treatment with 50 μM ECH remarkably attenuates the reduction of MMP, as Figure 1C shows. Fourthly, ECH protects against oxidative damage of mitochondrial protein in AC‐16 cells. As Figure 1D shows, the carbonyl protein content in mitochondria of ISO treatment cells is significantly increased and ECH attenuates the effect. Fifthly, ECH also inhibits mitochondrial lipid peroxidation in AC‐16 cells induced by ISO. As Figure 1E shows, the level of lipid peroxides in mitochondria of ISO cells is obviously higher than that of in Ctrl cells, while this increase is significantly reduced by 50μM ECH pre‐treatment. Ultimately, ECH inhibits the accumulation of intracellular ROS. As Figure 1F shows, the level of intracellular ROS in ISO cells is markedly increased and is prominently decreased in ECH pre‐treated cells. Figure 1G shows the apoptosis rate of AC‐16 cells is significantly increased following 24h incubations with 10μM ISO, comparing to the Ctrl cells, whereas pre‐treatment cells with 50 μM ECH significantly reduces ISO‐induced apoptosis.

### 3.2 ECH reverses myocardial remodelling and improves cardiac function of ISO‐induced HF rats

**Figure 2:**
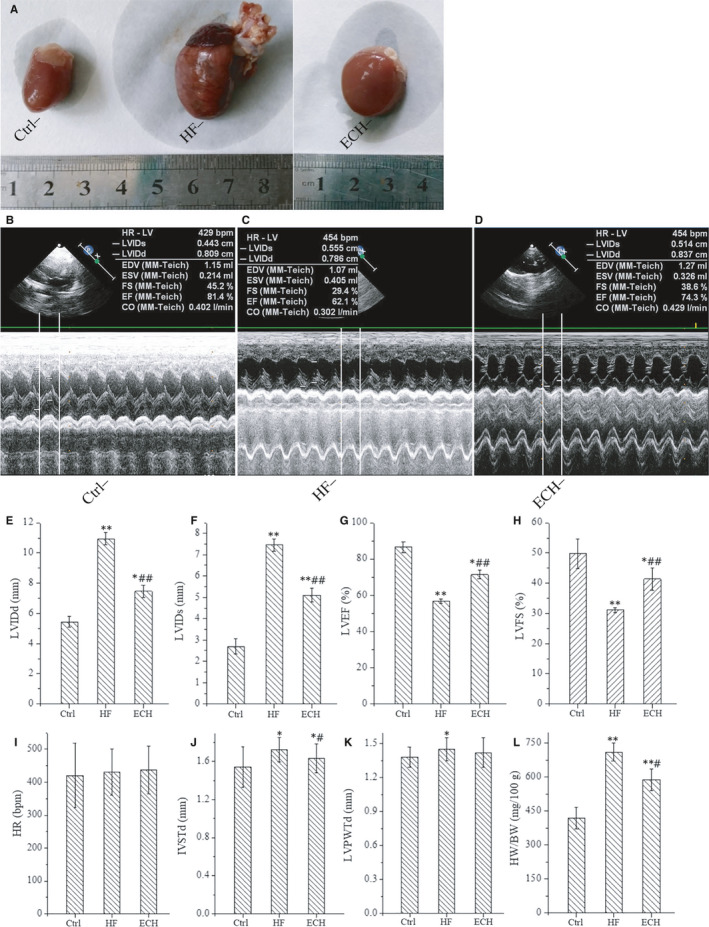
Cardiac hypertrophy of HF rats induced by ISO is relieved and heart function is significantly improved by ECH treated. A, ECH attenuates the morphological changes of rats. B‐D, Representative echocardiographic image of Ctrl, HF and ECH treated rat. E‐H, ECH effectively decreases LVIDd, LVIDs and increases LVEF, LVFS. I, There is no difference among three groups. J. ECH effectively decreases IVSTd. K, ECH has no effect in LVPWTd. L, ECH reduces the ratio of HW/BW. C‐E. F‐I.. All *P < .05 versus Ctrl,**P < .01 versus Ctrl; *^#^P* < .05 versus ISO, *^##^P* < .01 versus ISO. Error bars represent SD

As shown in Figure 2A, hearts in HF rats are obvious hypertrophy and is reversed by treatment with ECH. As Figure 2B‐D and L shows, ECH reduces the HW/BW. Echocardiography results show that LVEF and LVFS are significantly reduced in HF group, LVIDd, LVIDs and IVSTd are significantly increased in HF group. However, ECH significantly decreases LVIDd, LVIDs, IVSTd and improves LVEF and LVFS, there is no significant difference in HR among three groups, LVPWTd is increased in HF, but no difference between ECH and Ctrl, or between ECH and HF, all as Figure 2E‐K shows.

**Figure 3:**
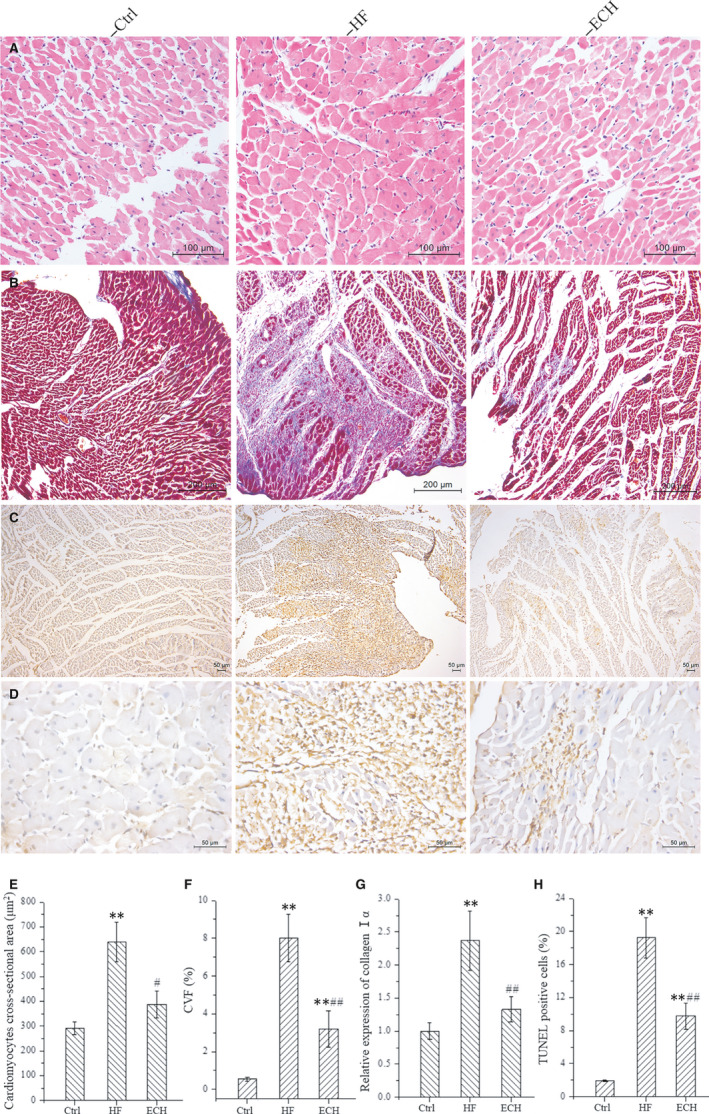
Results of histological staining demonstrate that ECH reverses left ventricular remodelling of HF rats induced by ISO. A, HE staining. B, Masson's Trichrome staining. C, TUNEL staining at low power. D, TUNEL staining at high power. E, ECH decreases cardiomyocyte cross‐sectional area. F, ECH reduces CVF. G, ECH reduces the mRNA expression of collagen Ⅰα. H, ECH inhibits myocardial apoptosis. All *P < .05 versus Ctrl,**P < .01 versus Ctrl; *^#^P* < .05 versus ISO, *^##^P* < .01 versus ISO. Error bars represent SD

Results of HE staining indicates that the myocardial hypertrophy, arranged disorder or degeneration necrosis, inflammatory cell infiltration and interstitial hyperaemia and oedema in HF rats, and ECH significantly alleviates these damages, myocardial hypertrophy and arranged disorder are relieved, the degeneration necrosis and inflammatory infiltrate are reduced, as shown in Figure 3A. The cardiomyocytes cross‐sectional area is measured, it is significantly increased after ISO and is decreased in ECH treated group, as Figure 3E shows. Assessments of collagen deposition by Masson staining reveals that left ventricular collagen content in HF rat is increased obviously and apparently decreased after treatment with ECH, which obviously decreases CVF. Real‐time PCR analysis reveals that procollagen type Ⅰ α transcripts are up‐regulated in ISO rat and down‐regulated in ECH rat, these results are consistent with each other, as Figure 3B and F‐G shows, the data indicate that ECH effectively reduces collagen deposition. The number of TUNEL‐positive cells in cardiomyocytes is significantly increased in HF rats compared with Ctrl, ECH markedly reduces myocardial cell apoptosis, and Figure 3C‐D shows the representative image of TUNEL‐positive cells in low and high power field respective, and Figure 3H shows the apoptosis rate of myocardial cells. These results indicate that ECH effectively reverses myocardial hypertrophy, myocardial interstitial fibrosis and apoptosis, and improves heart function in HF rat induced by ISO.

### 3.3 ECH protects myocardial ultrastructure in HF rats

**Figure 4:**
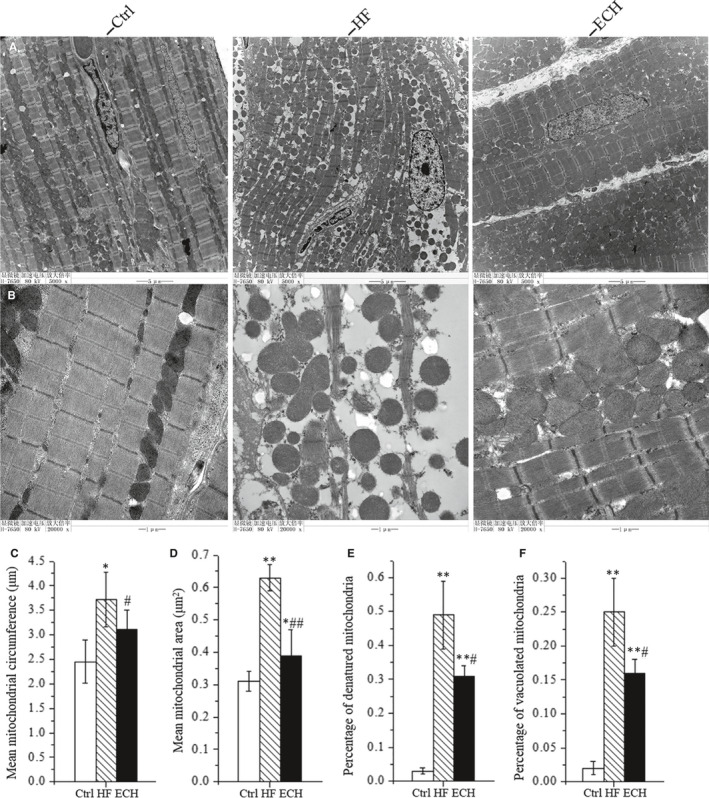
ECH protects the ultrastructure of cardiomyocytes and mitochondrial morphology of HF rats induced by ISO. A, Representative ultrastructural images of TEM at low power of Ctrl, HF and ECH rats. B, Representative images of TEM at high power of Ctrl, HF and ECH rats. C, ECH decreases mean mitochondrial circumference. D, ECH decreases mean mitochondrial cross‐sectional area. E, ECH reduces percentage of mitochondria degeneration. F. ECH reduces percentage of vacuolization in mitochondria. All *P < .05 versus Ctrl,**P < .01 versus Ctrl; *^#^P* < .05 versus ISO, *^##^P* < .01 versus ISO. Error bars represent SD

Transmission electron micrographs results show that the damage of myocardial ultrastructure could be attenuated by ECH, as Figure 4A‐B shows. Myofibrils are vertical arrangement, the sarcomere structure is obvious, the myofilaments are arranged neatly, and Z line is clear. Mitochondria are neatly arranged and the structure is clear in Ctrl rats. However, myofibrils are disorganized, ruptured and dissolved, the sarcomere structure is fuzzy, absence of the Z line, myocardial contraction zone is visible, the number and volume of mitochondria are increased, mitochondria are different shapes and sizes, arranged in disorder, structure are unclear, swelling and crista fragmentation, collapse or clumping and dissolved into vacuole in HF rats. ECH obviously attenuates these changes, the majority of the cardiac muscle fibres are neatly arranged and myocardial filaments are clear, Z line is existence, although some dissolution of filaments is apparent, the sarcomere structure is clear, the density of mitochondrial is decreased, the shapes and sizes are relieved, arranged more neatly, mitochondria swelling and crista fragmentation, collapse or clumping and vacuolization are evidently reduced by ECH compared with HF. Mitochondria swelling and crista fragmentation, collapse or clumping and partial vacuolization are identified as mitochondrial degeneration. The results demonstrate that ECH effectively protects myocardial ultrastructure, and especially protects mitochondrial morphology in HF rats, as Figure 4C‐F show.

### 3.4 ECH up‐regulates protein expression of SIRT1, FOXO3a and MnSOD

**Figure 5:**
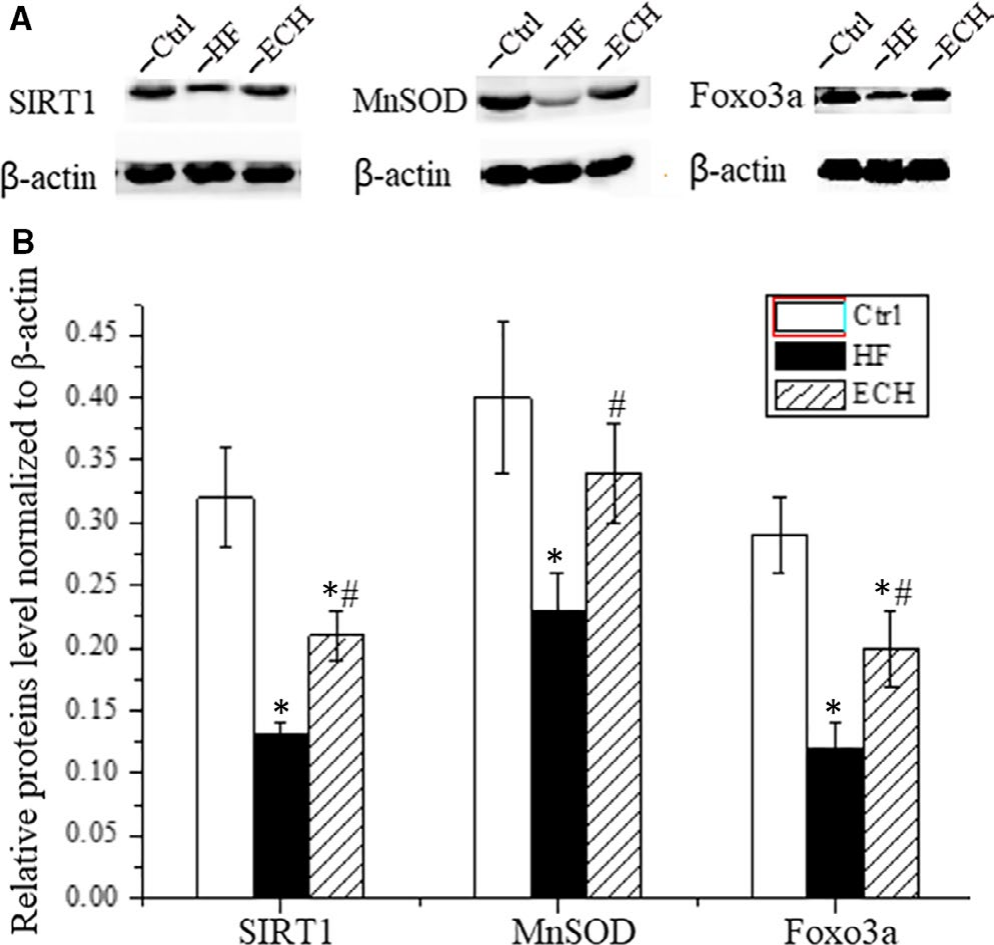
ECH up‐regulates the protein expression level of SIRT1, FOXO3a and MnSOD which down‐regulated in HF rats. A, Representative bands of western blotting of SIRT1, FOXO3a and MnSOD of Ctrl, HF and ECH rats. B, Statistical histogram of protein expression level of SIRT1, FOXO3a and MnSOD. All *P < .05 versus Ctrl,**P < .01 versus Ctrl; *^#^P* < .05 versus ISO, *^##^P* < .01 versus ISO. Error bars represent SD

Western Blot analysis demonstrates that the protein expression level of SIRT1, FOXO3a and MnSOD are significantly down‐regulated in HF rat, however, ECH obviously up‐regulates the decreased protein expression. The representative protein bands of immunoblotting and statistical analysis results are showed in Figure 5. The part of the data indicates that ECH increases the protein expression of SIRT1, FOXO3a and MnSOD.

### 3.5 ECH inhibits mitochondrial oxidative stress in left ventricular myocardial tissue

**Figure 6:**
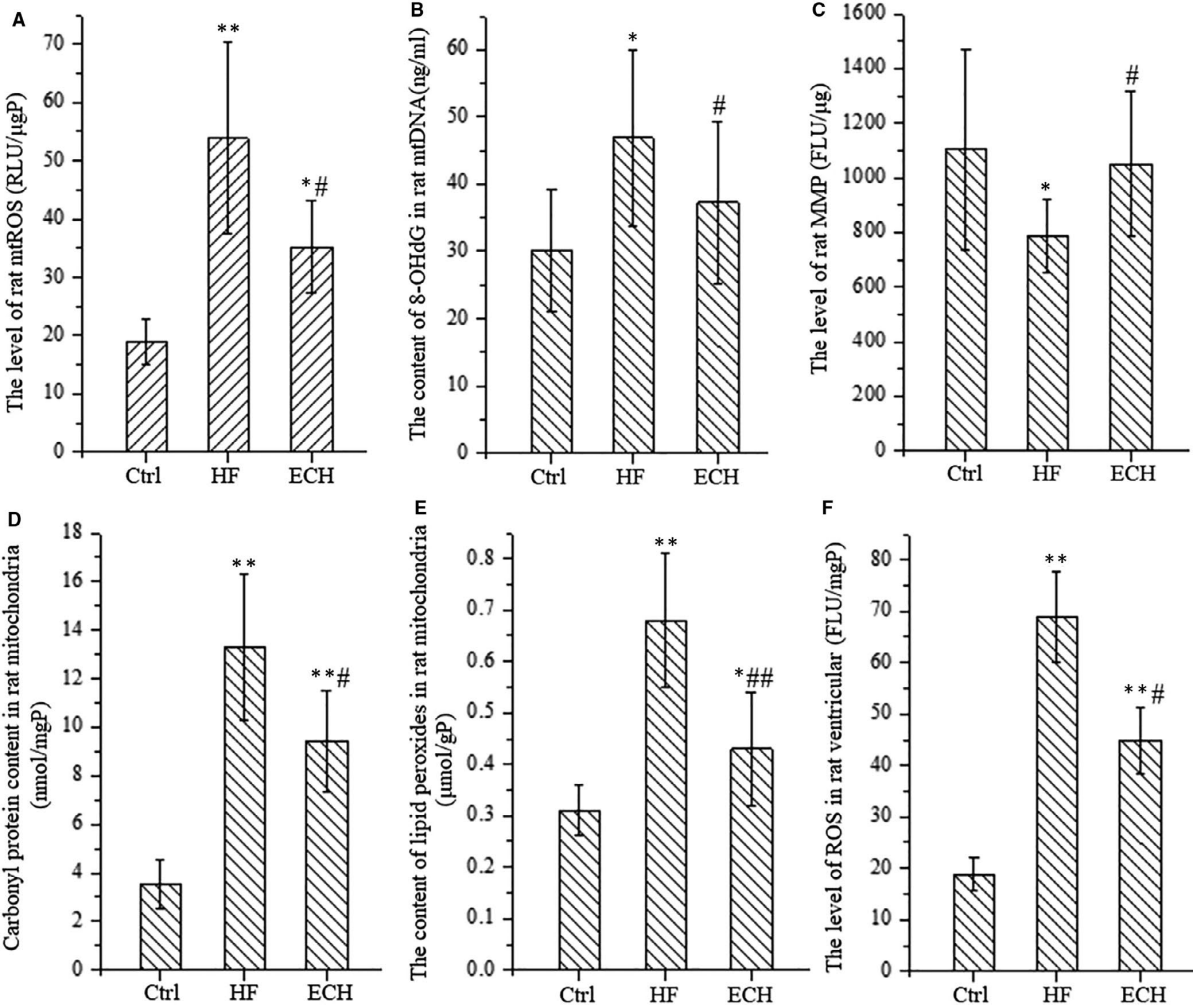
ECH significantly inhibits mitochondrial oxidative stress in left ventricular tissue of HF rats induced by ISO and protects mitochondrial function. A, ECH reduces the release of mitochondrial ROS which enhanced in HF, y represents the relative light unit (RLU) per μg mitochondrial protein. B, ECH inhibits oxidative damage of mtDNA which increased in HF, y represents the level of 8‐OHdG. C, ECH protects MMP which damaged in HF, y represents the fluorescence intensity per μg mitochondrial protein. D, ECH decreases carbonyl protein content in mitochondria, which increased in HF, y represents the level of carbonyl protein per mg mitochondrial protein. E, ECH decreases lipid peroxidation level in mitochondria, which increased in HF, y represents the level of lipid peroxides MDA per g mitochondrial protein. F. ECH reduces the accumulation of ROS in left ventricular tissue of rats, which increased in HF. All *P < .05 versus Ctrl,**P < .01 versus Ctrl; *^#^P* < .05 versus ISO, *^##^P* < .01 versus ISO. Error bars represent SD

In this part, mitochondrial ROS, MMP, oxidative damage of mitochondrial DNA, carbonyl protein content and lipid peroxidation level in mitochondria in left ventricular myocardial tissue of rats are all measured, and the results are consistent with cell experiments, that is to say, ECH decreases mitochondrial ROS, protects MMP, relieves the damage degree of mtDNA, reduces carbonyl protein content and lipid peroxidation in mitochondrial, as Figure 6 shows.

## 4 DISCUSSION

This study demonstrates that ECH exerts effective roles in the prevention of myocardial remodelling and improvement of heart function via up‐regulating SIRT1/FOXO3a/MnSOD signalling axis and inhibiting mitochondrial oxidative stress.

Mitochondria are major site of generation of ROS as a by‐product of oxidative phosphorylation, by their proximity to ROS, mitochondrial proteins, lipids and mtDNA are believed to be primary targets of oxidative damage after excessive emission of mitochondrial ROS,^34^, ^35^ this damage stimulates further production of ROS, the vicious circle continues, ROS and oxidative stress could led to myocardial hypertrophy, apoptosis, interstitial fibrosis and HF through activation of multiple signalling pathways.^35^ So, it seems that drugs targeted inhibition of mitochondrial oxidative stress are effective for the prevention of myocardial remodelling. ECH is a natural phenylethanoid glycoside, a component of traditional chinese herb *Cistanche tubulosa*. it is widely studied and has been proved possessing kinds of pharmacological activities in recent years, such as anti‐tumour, anti‐apoptosis, anti‐ageing, neuroprotective action, and so on. Studies have proved that the pharmacological actions of ECH mainly depend on its property of antioxidant and anti‐inflammatory activity,^18^, ^21^ and ECH decreases mitochondrial ROS and protects mitochondrial function against oxidative damage in nerve cells.^20^, ^22^ Here, we firstly demonstrated that ECH decreases mitochondrial ROS of cardiomyocytes, hence, improves MMP, reduces oxidative damage of mtDNA, protein and lipids, effectively inhibits mitochondrial oxidative damage and reduces apoptosis in vitro and in vivo, so, it reverses myocardial remodelling and improves heart function. It means that ECH is a potential new drug for prevention of myocardial remodelling and HF.

In normal conditions, mitochondrial ROS is mainly cleared by MnSOD which distributes in mitochondria and protects against mitochondrial oxidative damage. Ang II significantly decreases MnSOD expression of cardiomyocytes and induces myocardial hypertrophy, however, resveratrol could increase the expression of MnSOD and inhibit myocardial hypertrophy induced by Ang II.^36^ In HF, the activity of MnSOD is significantly decreased and the generation of mitochondrial ROS is elevated.^37^, ^38^ In cardiac specific MnSOD gene knockout mice, mitochondrial ROS could not be cleared and ROS is increased in cardiomyocytes, this led to mitochondrial dysfunction and progressive cardiac enlargement, eventually lead to heart failure.^39^ In present study, we confirm that the expression of MnSOD is significantly decreased, which is attenuated by ECH treatment, and thus, mitochondrial ROS is reduced and mitochondrial oxidative stress is inhibited. It means that ECH protects cardiomyocytes against oxidative damage through up‐regulation of the expression of MnSOD, we further explore the molecular mechanisms underlying these effects.

The expression of MnSOD is regulated by FOXO3a, which is a transcription factor and binds in the gene promoter region of MnSOD and leads to the promotion of gene transcription.^40^ SIRT1 is an upstream molecular of FOXO3a and it can activate FOXO3a by deacetylation. SIRT1/FOXO3a/MnSOD signalling axis plays pivotal roles in protection against oxidative stress in kinds of cell types, activation of the signalling axis by estradiol inhibits oxidative stress and exerts the cardiac protection^24^; Activation the signalling axis by resveratrol inhibits oxidative stress induced cell death^40^; Otherwise, angiotensin II inhibits SIRT1/FoxO3a/MnSOD pathway and induces mitochondrial oxidative stress, mtDNA damage in osteoblasts,^41^ type 5 adenylyl cyclase increases oxidative stress by inhibition on the expression of MnSOD via the Sirt1/FoxO3a Pathway.^42^ The present study finds that the expression of SIRT1, FoxO3a and MnSOD all are significantly reduced in HF rats and mitochondrial ROS is elevated and oxidative stress is enhanced, however, the changes are reversed by ECH. New research indicates that ECH directly binds SIRT1 as a ligand and forms a complex with 9 covalent bonds and up‐regulates the expression of SIRT1 in nerve cells.^26^ Consistent with this study, the present study demonstrates that ECH also up‐regulates the expression of SIRT1 in cardiomyocytes, it then up‐regulates gene expression of MnSOD through FoxO3a and inhibits mitochondrial ROS and oxidative stress.

**Figure 7:**
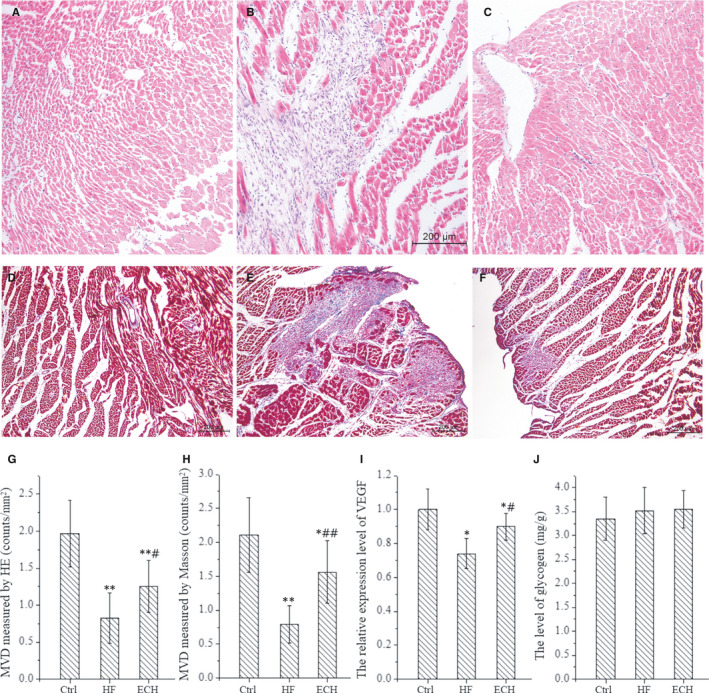
The effects of ECH in the expression of VEGF, MVD and glycogen levels. A‐C, Representative images of microvessels using HE staining. D‐F, Representative images of microvessels using Masson staining. G, Statistical histogram of MVD using HE Masson staining. H, Statistical histogram of MVD using Masson staining. I, The expression of VEGF in ventricular tissue of rats. J, Glycogen levels in ventricular tissue of rats. All *P < .05 versus Ctrl,**P < .01 versus Ctrl; *^#^P* < .05 versus ISO, *^##^P* < .01 versus ISO. Error bars represent SD

Long‐term chronic ischaemia can cause myocardial hibernation, these hibernating myocardial cells typically show a loss of contractile filaments, there are excess glycogen granules in the cytosol, sarcoplasmic reticulum and transverse tubules are lost,^43^ also, there are abnormalities in mitochondrial size and shape, reactive myocardial hypertrophy and interstitial fibrosis, which secondary to myocyte loss.^44^ We also observed the above changes in HF rats in our study, whereas ECH significantly relieves these changes, as the latest study reported recovery of cardiac function after ischaemic stress may be a feature of myocardial hibernation,^45^ the myocardial glycogen level is also measured, the results indicates that there is no significant difference among the three groups, as shown in Figure 7J.

The previous study also detected myocardial hibernation in a non‐ischaemic heart failure pig model by pacing the LV free wall with high‐frequency,^46^ because microvascular obstruction plays important roles in myocardial hibernation and the resulting contractile dysfunction, and that are marked improved when the microvascular obstruction is reversed by drug.^47^ At present, some study conclude that revascularization therapy is effective to hibernating myocardium,^43^ by using HE and Masson staining, we observe that myocardial capillary and arteriolar density are reduced obviously in HF rats and are relieved significantly by ECH, as shown in Figure 7A‐H. Previous studies have demonstrated that other natural compounds, such as barley beta glucan exert cardioprotective effects by increasing expression of MnSOD,^48^, ^49^ and rising of MnSOD is related to increased myocardial capillary density and VEGF expression. We performed RT‐PCR to detected the expression of VEGF, the data are consistent with the finding of histological staining, it means that ECH may promote angiogenesis and increase myocardial capillary density via regulating VEGF expression, as shown in Figure 7I. The present study finds that ECH can protect and increase myocardial capillary and arteriolar via up‐regulating SIRTI/FOXO3a/MnSOD pathway and the expression of VEGF, thereby suppressing hibernating myocardium and contributing to improve heart function.

Of note, cardiomyocyte apoptosis is well known to be an important pathologic changes in heart failure, and the present study indeed confirms that large amount of apoptotic cells in HF rats and ECH significantly reduces the apoptosis via regulating SIRTI/FOXO3a/MnSOD pathway and inhibiting mitochondrial oxidative stress, as mentioned above.

Above all, ECH reverses myocardial remodelling and improves heart function via up‐regulating SIRT1/FoxO3a/MnSOD signalling axis, and thus inhibits mitochondrial oxidative stress and reduces hibernating myocardium. Thus, it is suggested that ECH is a potential drug for prevention or treatment of myocardial remodelling and HF.

## CONFLICT OF INTEREST

The authors declare that they have no competing interests.

## AUTHOR CONTRIBUTIONS

JW‐Z and YJ‐N designed the study, planned and performed experiments, analysed data and drafted and revised the manuscript. JD, XL and QL helped to perform experiments, administrated experiments, collected and analysed data. JL‐Z validated data, supported software and revised the manuscript.

## Supporting information

Supplementary Material

## Data Availability

The data used to support the findings of this study are available from the corresponding author upon request.
