# Supplementary material for: Echinacoside reverses myocardial remodeling and improves heart function via regulating SIRT1/FOXO3a/MnSOD axis in HF rats induced by isoproterenol
Source: J Cell Mol Med. 2020 Dec 13;25(1):203–16. doi: 10.1111/jcmm.15904 (PMC7810933; doi:10.1111/jcmm.15904)
Supplement: Supplementary file 1 — Supplementary Material [file JCMM-25-203-s001.docx]

**Supplementary Methods**

**Detection of glycogen levels in ventricular**

Myocardial glycogen level is measured with the anthracenone method. About 100mg left ventricular tissue is cutted from frozen heart tissue and maked homogenate in 0.75ml extracting solution, the boiled in 95℃ water for 20min, mixed by oscillating every five minutes, they are cooled after all the tissues have dissolved and add water to 5ml, centrifuged at 8000r/min, 25℃, 10min, the supernatant is detected according to the manufacturer’s instructions. The OD is measured in spectrophotometer at the wavelength of 620nm, and converted into mg/g myocardial tissue.

**Detection of** **VEGF expression level**

Quantitative PCR is used to detected the VEGF expression level, as described in Materials and Methods. Specific primer sequences of collagen I used for real-time PCR is as follows: forward: 5′-AACCTCACCAAAGCCAGCAC-3′, revers: 5′-AACCGGGATTTCTTGCGCTT-3′.

**Measurement of MVD**

By using HE and Masson staining, the MVD is measured. Image-pro Plus 6.0 is used, six fields are randomly selected under 200X microscope, and the number of microvessels in the field is counted and averaged, and converted into counts/mm^2^.
